# Detection of pulmonary embolism severity using clinical characteristics, hematological indices, and machine learning techniques

**DOI:** 10.3389/fninf.2022.1029690

**Published:** 2022-12-16

**Authors:** Hang Su, Zhengyuan Han, Yujie Fu, Dong Zhao, Fanhua Yu, Ali Asghar Heidari, Yu Zhang, Yeqi Shou, Peiliang Wu, Huiling Chen, Yanfan Chen

**Affiliations:** ^1^College of Computer Science and Technology, Changchun Normal University, Changchun, Jilin, China; ^2^Department of Pulmonary and Critical Care Medicine, The First Affiliated Hospital of Wenzhou Medical University, Wenzhou, China; ^3^School of Surveying and Geospatial Engineering, College of Engineering, University of Tehran, Tehran, Iran; ^4^College of Computer Science and Artificial Intelligence, Wenzhou University, Wenzhou, Zhejiang, China

**Keywords:** pulmonary embolism, feature selection, extreme learning machine, disease diagnosis, machine learning, meta-heuristic, swarm-intelligence

## Abstract

**Introduction:**

Pulmonary embolism (PE) is a cardiopulmonary condition that can be fatal. PE can lead to sudden cardiovascular collapse and is potentially life-threatening, necessitating risk classification to modify therapy following the diagnosis of PE. We collected clinical characteristics, routine blood data, and arterial blood gas analysis data from all 139 patients.

**Methods:**

Combining these data, this paper proposes a PE risk stratified prediction framework based on machine learning technology. An improved algorithm is proposed by adding sobol sequence and black hole mechanism to the cuckoo search algorithm (CS), called SBCS. Based on the coupling of the enhanced algorithm and the kernel extreme learning machine (KELM), a prediction framework is also proposed.

**Results:**

To confirm the overall performance of SBCS, we run benchmark function experiments in this work. The results demonstrate that SBCS has great convergence accuracy and speed. Then, tests based on seven open data sets are carried out in this study to verify the performance of SBCS on the feature selection problem. To further demonstrate the usefulness and applicability of the SBCS-KELM framework, this paper conducts aided diagnosis experiments on PE data collected from the hospital.

**Discussion:**

The experiment findings show that the indicators chosen, such as syncope, systolic blood pressure (SBP), oxygen saturation (SaO2%), white blood cell (WBC), neutrophil percentage (NEUT%), and others, are crucial for the feature selection approach presented in this study to assess the severity of PE. The classification results reveal that the prediction model’s accuracy is 99.26% and its sensitivity is 98.57%. It is expected to become a new and accurate method to distinguish the severity of PE.

## Introduction

A potentially fatal cardiac condition known as pulmonary embolism (PE) occurs when one or more emboli clog the pulmonary artery, impairing breathing and pulmonary circulation (Goldhaber, 2004). Tumor, fat, amniotic fluid, or air can all cause PE. The most prevalent cause of pulmonary embolism, however, is deep vein thrombosis (DVT), a blood clot that most usually occurs in the deep veins of the lower legs. 53–162 DVT cases per 100,000 persons are reported annually, according to epidemiological studies. The yearly incidence of PE ranges from 39 to 115 per 100,000 people (Wendelboe and Raskob, 2016; Keller et al., 2020). In addition, PE claims about 300,000 lives annually in the US (Wendelboe and Raskob, 2016; Konstantinides et al., 2020).

The clinical symptoms of pulmonary embolism are non-specific. The typical clinical presentation of PE includes dyspnea, chest pain, hemoptysis, presyncope or syncope, collapse, hypotension, right heart failure, cardiogenic shock, and sudden cardiac death (Hirsh et al., 1986; Keller et al., 2016a,b). For young and previously healthy patients with excellent cardiac reserve, PE can also be asymptomatic (Goldhaber, 2004). The non-specific and variable clinical manifestations of PE, which challenge the diagnosis and treatment, are mainly related to the patient’s hemodynamic status and right ventricular load (Agnelli and Becattini, 2015).

Pulmonary embolism can take the form of small, asymptomatic blood clots or big, life-threatening emboli that block the pulmonary arteries and cause a rapid circulatory collapse. After diagnosing PE, risk stratification must be done in order to modify the course of treatment since PE poses a possible hazard to life (Konstantinides and Goldhaber, 2012). The optimum method for risk classification of patients with PE is currently that suggested by the European Cardiology Society (ESC) (Jen et al., 2018; Wang et al., 2019; Konstantinides et al., 2020). However, certain investigations continue to demonstrate that more than 50% of patients with acute PE remain hemodynamically stable according to clinical models, although there is a risk of mortality (Elias et al., 2016; Barrios et al., 2017). Another research investigated the 2014 ESC model’s capacity to forecast mortality 30 days after acute PE, demonstrating the need for additional development in the categorization of intermediate-risk patients (Becattini et al., 2016). Therefore, it is crucial to develop new techniques for more precise risk assessment of individuals with PE. In recent years, the use of machine learning techniques to aid physicians in resolving medical problems has increased.

By fusing the Chaotic Emperor Penguin Optimization (CEPO) algorithm with an Extreme Learning Machine, Baliarsingh and Vipsita (2020) proposed a cancer categorization prediction model with good accuracy and sensitivity. Ahmadi and Afshar (2016) introduced the traditional Particle Swarm Optimization algorithm into a Support Vector Machine (SVM) to achieve more accurate classification prediction of breast cancer. Mishra et al. (2021) used machine learning techniques to segment MRI brain images and improved the model’s accuracy using a whale optimization algorithm. For a brand-new ECG arrhythmia classification issue, Khazaee and Zadeh (2014) suggested a SVM classifier model based on particle swarm optimization (PSO). In order to forecast Parkinson’s illness, Cai et al. (2017) suggested an ideal SVM based on bacterial foraging optimization (BFO). They experimentally confirmed that the improved technique has a high classification accuracy. To control the nutritional cycle in hospitals, Ileri and Hacibeyoglu (2019) suggested a hybrid metaheuristic machine learning model that combines genetic algorithms, simulated annealing algorithms, and machine learning techniques. Zhou et al. (2014) used a Memetic algorithm to optimize chained weight vectors and combined it with an extreme learning machine for classifying metabolite features. Lambert and Perumal (2021) developed a new meta-heuristic classification model for the diagnosis and optimal feature selection of chronic kidney disease, and the proposed model achieved a high accuracy rate. Many medical diagnosis systems can assist doctors in making more intelligent and successful decisions (Li et al., 2022; Liu et al., 2022a,d). An increasing number of researchers can be seen to be using machine learning classification prediction techniques for medical diagnosis in recent years. Traditional feature selection methods are highly prone to data overlap when there are not enough features, which will lead to classifier failure in this case. In addition, when the dimensionality of features is too high, the distance of similar data in the space becomes sparse, which also decreases the efficiency and accuracy of the classifier. The feature selection model based on the swarm intelligence algorithm is based on global feature selection, which is less likely to overlap when the amount of data is small. On the other hand, the swarm intelligence algorithm can take advantage of its stochastic and collaborative nature when dealing with high-dimensional data and can produce high-quality solutions and high-precision classification results in a limited amount of time.

Most traditional optimizers need to deal with info related to the surface of the feature space, or they need an offline routine to deal with problems (Zhang M. et al., 2021). A fresh approach for resolving these issues is the swarm intelligence optimization algorithm, which is well-liked by academics due to its effectiveness and great precision. The swarm intelligence optimization technique is developed by abstractly modeling the cooperative behavior of animals, fish, insects, and other natural entities. For example, there are different evolution (DE) (Storn and Price, 1997), chaotic BA (CBA) (Adarsh et al., 2016), sine cosine algorithm (SCA) (Mirjalili, 2016), salp swarm algorithm (SSA) (Mirjalili et al., 2017), whale optimizer (WOA) (Mirjalili and Lewis, 2016), moth-flame optimization (MFO) (Mirjalili, 2015), hunger games search (HGS) (Yang et al., 2021), Harris hawks optimization (HHO) (Heidari et al., 2019), slime mold algorithm (SMA) (Li et al., 2020), moth-flame optimizer with sine cosine mechanisms (SMFO) (Chen et al., 2021), colony predation algorithm (CPA) (Tu et al., 2021), the weighted mean of vectors (INFO) (Ahmadianfar et al., 2022), Runge Kutta optimizer (RUN) (Ahmadianfar et al., 2021), particle swarm optimization (PSO) (Kennedy and Eberhart, 1995), fruit fly optimization algorithm (FOA) (Pan, 2012), improved ant colony optimizer (RCACO) (Zhao et al., 2020), improved WOA (EWOA) (Tu et al., 2020), chaotic SCA (Ji et al., 2020), and so on. They have also been used in many fields, such as image segmentation (Hussien et al., 2022; Yu et al., 2022), optimization of machine learning models (Chen et al., 2014), scheduling problems (Gao et al., 2020; Han et al., 2021; Wang G. G. et al., 2022), feature selection (Hu J. et al., 2022; Liu et al., 2022b), complex optimization problem (Deng et al., 2022b), bankruptcy prediction (Xu et al., 2019; Zhang Y. et al., 2021), resource allocation (Deng et al., 2022a), gate resource allocation (Deng et al., 2020a,b), airport taxiway planning (Deng et al., 2022c), robust optimization (He et al., 2019, 2020), solar cell parameter Identification (Ye et al., 2021), and medical diagnosis (Chen et al., 2016; Wang et al., 2017).

Yang and Suash (2009) proposed a novel and high-performance evolutionary algorithm called cuckoo search (CS) algorithm by simulating the parasitic collaborative behavior of cuckoos. It has been widely used by researchers in various fields of optimization problems due to its high exploration and exploitation capabilities. Jin et al. (2015) proposed a high search efficiency CS algorithm for designing PID controllers. Boushaki et al. (2018) introduced the quantum chaos mechanism into the CS algorithm to improve the convergence speed of the algorithm and used the improved algorithm in data clustering. Zhou et al. (2013) developed a CS algorithm combining three strategies for solving the planar graph coloring problem. Valian et al. (2013) improved the convergence accuracy and speed by adjusting the parameters of the CS algorithm and applying the algorithm to engineering optimization problems. Sheng et al. (2014) proposed an adaptive CS algorithm for optimizing the parameters of chaotic systems. Since no optimization algorithm can optimize all different types of problems, the original CS algorithm is no exception. The CS algorithm has low search breadth in the first iteration and it is easy to fall into local optimum in the process of optimal finding. The CS algorithm is prone to slow search speed and poor convergence accuracy when applied to optimize feature selection models.

As a result, a new and better version of the CS algorithm (SBCS) is proposed in this paper, which combines the sobol sequence and the black hole mechanism with the original algorithm to improve its optimization capability. This study performs benchmark experiments employing 30 CEC 2014 functions to validate the algorithm performance of SBCS. The SBCS algorithm is compared with four original algorithms and four improved algorithms. To further verify that the SBCS-KELM model is more competitive, a series of validation experiments are conducted for this model on real hospital datasets (PE), mainly including the comparison experiments of five classical machine learning classification algorithms based on SBCS, the comparison experiments of SBCS-KELM with other famous classifiers and the comparison experiments of 10 feature selection models based on group intelligence optimization algorithm and KELM. Furthermore, we successfully demonstrate the superior competitiveness of the proposed SBCS-KELM model by analyzing the results of the above three comparative experiments using the following four evaluation metrics: Accuracy, Sensitivity, F-measure, and MCC. Finally, we discuss the five key features obtained from the results of 10 time 10-fold cross-validation experiments based on a medical perspective to prove that the results align with the actual statistical significance in this paper.

The main contributions of this study can be summarized as: (1) An effective aid to the diagnosis of pulmonary embolism is proposed. (2) A KELM model is developed based on an improved swarm intelligence optimization algorithm. (3) The SBCS algorithm is proposed, while it is an improved strong performance swarm intelligence optimization algorithm.

The remainder of this paper is structured as follows. In section “Materials and methods,” this paper describes the materials used and the CS algorithm. In section “The proposed method,” the paper presents the improved SBCS algorithm and the SBCS-KELM model. In section “Experimental results and discussions,” the paper experimentally validates the core advantages of the SBCS algorithm and the SBCS-KELM model. In section “Discussions,” the paper combines practical medical knowledge and experimental results for a detailed discussion. Finally, in section “Conclusions and future works,” the paper is summarized and looks to the future.

## Materials and methods

In this section, the source of the PE dataset and its acquisition criteria are first described. Then this section describes the original cuckoo (CS) algorithm, including its core idea, formulas, and pseudo-code.

### Pulmonary embolism data collection

Data from pulmonary embolism patients hospitalized to the First Affiliated Hospital of Wenzhou Medical University between April 2014 and May 2020 were retrospectively gathered for this single-center study. The diagnosis of PE meets at least one of the following criteria: (1) confirmed by computed tomographic pulmonary angiography (CTPA). (2) Confirmed by pulmonary perfusion imaging. (3) Clinical diagnosis: need to meet the following conditions: (1) having typical clinical manifestations. (2) DVT confirmed by lower extremity vascular ultrasound. (3) D-Dimer > 0.5 mg/L. The 139 PE patients were divided into two groups: the intermediate-low-risk group (*n* = 70) and the high-risk group (*n* = 69). According to the definition of ESC guidelines and the American Heart Association (AHA) scientific statement, PE patients with a systolic blood pressure < 90 mmHg are classified as a high-risk group (Jaff et al., 2011; Konstantinides et al., 2020).

All 139 patients’ clinical details, blood test results, and information on arterial blood gas analysis were recorded. The blood samples were taken three days after the diagnosis of PE. Table 1 contains a list of the data. SPSS statistics 24.0 was used to conduct the statistical analysis. The chi-square test was used to assess categorical variables. Independent sample *t*-test was used to assess continuously varying variables. Statistics consider something significant if *p* < 0.05. The findings of the precise statistical analysis are displayed in Tables 2, 3.

**TABLE 1:** Numbered list of the characteristics utilized in this study and their meanings.

	Features	Abbreviation
**F1**	**Age**	**Age**
F2	Gender	Gender
F3	Dyspnea	Dyspnea
F4	Chest pain	CP
F5	Hemoptysis	Hemoptysis
F6	Syncope	Syncope
F7	Cardiopulmonary resuscitation	CPR
F8	Altered mental status	AMS
F9	Chronic heart failure	CHF
F10	Chronic lung disease	CLD
F11	History of tumor	HOT
F12	Systolic blood pressure	SBP
F13	Diastolic blood pressure	DBP
F14	Pulse rate	PR
F15	Temperature	T
F16	Respiratory rate	RR
F17	White blood cell	WBC
F18	Neutrophil percentage	NEUT%
F19	Hemoglobin	HGB
F20	Blood platelet	PLT
F21	Hydrogen ion concentration	PH
F22	Oxygen saturation	SaO_2_%
F23	Right heart dysfunction	RHD

**TABLE 2:** Clinical characteristics in intermediate-low-risk PE patients and high-risk PE patients.

Index	Intermediate-low-risk PE (*n* = 70)	High-risk PE (*n* = 69)	χ^2^ value	*p*-value
Gender (Male/Female)	40/30	30/39	2.595	0.107
Dyspnea (No/Yes)	30/40	26/43	0.387	0.534
CP (No/Yes)	57/13	61/8	1.319	0.251
Hemoptysis (No/Yes)	66/4	67/2	0.159	0.689
Syncope (No/Yes)	67/3	49/20	15.351	0.001
CPR (No/Yes)	70/0	61/8	6.607	0.010
AMS (No/Yes)	66/4	55/14	6.549	0.011
CHF (No/Yes)	57/13	55/14	0.066	0.798
CLD (No/Yes)	56/14	61/8	1.843	0.175
HOT (No/Yes)	59/11	47/22	5.018	0.025
RHD (No/Yes)	57/13	51/18	1.133	0.287

**TABLE 3:** Blood routine, arterial blood gas analysis and clinical parameters in intermediate-low-risk PE patients and high-risk PE patients.

Index	Intermediate-low-risk PE (*n* = 70)	High-risk PE (*n* = 69)	*p*-value
Age	65.30 ± 13.380	64.65 ± 11.732	0.762
SBP	119.00 ± 21.404	86.90 ± 8.168	0.000
DBP	70.64 ± 12.059	54.51 ± 9.646	0.000
PR	89.49 ± 14.589	92.13 ± 25.532	0.456
T	37.16 ± 0.578	37.20 ± 1.291	0.833
RR	20.11 ± 2.579	21.09 ± 5.412	0.180
WBC	8.40 ± 4.866	10.36 ± 6.128	0.039
NEUT%	0.67 ± 0.135	0.73 ± 0.142	0.012
HGB	124.63 ± 20.391	112.13 ± 25.903	0.002
PLT	232.67 ± 137.808	216.57 ± 115.283	0.456
PH	7.42 ± 0.039	7.41 ± 0.081	0.736
SaO_2_%	95.52 ± 3.068	92.69 ± 11.078	0.044

### Description of cuckoo search algorithm

The mathematical model, pseudo-code and flowchart of the CS algorithm are described in detail in this subsection.

#### Mathematical model

The cuckoo search algorithm (CS) is an optimization algorithm that draws on the behavior of cuckoos in finding nest locations to find eggs to lay. The cuckoo does not make a nest nor does it brood. Before laying eggs, it pushes the eggs of the host bird out of the nest when the other bird (host bird) leaves the nest and lays its own eggs in the host’s nest, allowing the host bird to feed the cuckoo chicks. The juvenile cuckoos, which are raised by the host bird, also have the habit of pushing the host bird’s young out of the nest, and will mimic the behavior to reduce the probability of being detected by the host bird.

Assume that the cuckoo search algorithm satisfies the following three idealized conditions:

(1) Cuckoos randomly select a suitable nest to lay one egg at a time.
(2) The best nest from the group of nests chosen at random will be kept for the following generation.
(3) The number of nests that can be used is fixed, and the probability that the owner of a nest can find an alien egg, also known as *Pa*∈[0,1].

The algorithm location update formula is as follows:


(1) $$x_{i}\,\left(t+1\right)=x_{i}\,\left(t\right)+\alpha \,⊗L\,e\,v\,y\,\left(\beta \right)$$

where *x*_*i*_(*t* + 1) denotes the nest position of the *i*th nest at generation *t*; ⊗ is the inner product notation, which indicates the multiplication of vectors; α denotes the step control factor, and *Levy*(β) is the Levy random search path.


(2) $$\text{Lévy}∼\mu =t^{-1-\beta },0<\beta \leq 2$$

After updating the nest location, the adaptation values of the nests are calculated and compared, and the solution with better adaptation is selected. After that, an equal number of new solutions are generated by discarding some of the poor solutions according to the probability *Pa* and using a biased random walk.


(3) $$x_{i}\,\left(t+1\right)=x_{i}\,\left(t\right)+r\,⊗\mathrm{Heaviside}\,\left(P_{a}-\varepsilon \right)\,⊗\left(x_{k}\,\left(t\right)-x_{j}\,\left(t\right)\right)$$

where *r*,ε ∈ [0,1] are normally distributed random numbers; Heaviside(*u*) is the unit transitive function, which is the step function; *x*_*k*_(*t*),*x*_*j*_(*t*) are the different random solutions in the *t*th generation. The contemporary optimal solution and the associated fitness value are preserved after one population iteration is finished. The method above is then repeated until the maximum number of iterations is achieved, at which point the global optimal solution is produced.

#### The pseudo-code and flowchart

**FIGURE 1:**
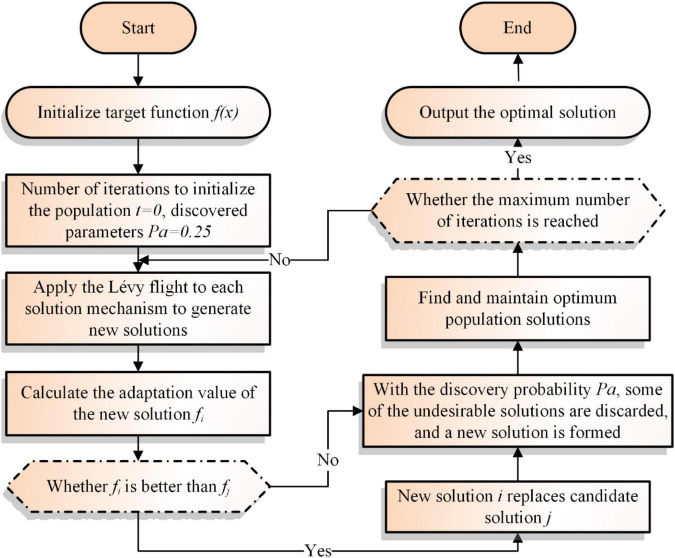
Flowchart of CS.

In this section, the pseudo-code and flowchart for CS is illustrated as shown in Algorithm 1 and Figure 1 respectively.

**Algorithm 1:** Pseudo-code of CS.

Initialize the fitness value function *f*(*x*),*x* = (*x*_1_,*x*_2_,*x*_3_,⋯,*x*_*d*_)*T* Initialize the number of iterations*t* = 0, discovered parameters *P*_*a*_ = 0.25 Initialize the individual solution $x_{i}^{0}$ of the overall *N* solutions, (*i* = 1,2,…,*N*) **While** *l* ≤ *Maximum number of iterations* Using Lévy flight Update all search agents $x_{i}^{t}$ in the population Evaluate the new solution *x^t^*_*new,i*_ for its fitness value *f^t^*_*new,i*_ **If** the new solution has a better fitness value Replace the old solution $x_{j}^{t}$ with the updated solution *x^t^*_*new,i*_ **End If** Discard some poor-quality solutions by probability *P_a_* and replace them with random ones Increase in the number of iterations *t* = *t* + 1 **End While** Return the best solution

## The proposed method

We first introduce the sobol sequence and the black hole mechanism and propose the SBCS algorithm by adding these two strategies to the CS algorithm. Further, this section introduces the discretization strategy and the KELM classifier and proposes the SBCS-KELM prediction model by combining the SBCS algorithm.

### The proposed SBCS

#### Sobol sequence

The distribution of starting populations has a significant impact on the algorithm’s accuracy and speed of convergence in metaheuristic algorithms. To achieve high traversal and variety while solving issues with uncertain distributions, the starting population values should be dispersed over the search space as equally as feasible. The cuckoo search algorithm generates initialized populations in the search space using random numbers. This method has low traversal and unpredictable distribution of individuals.

Low discrepancy sequences, commonly known as the suggested Monte Carlo approach, employ deterministic low discrepancy sequences as opposed to pseudo-random sequences (QMC). QMC offers greater efficiency and uniformity in dealing with probabilistic problems by choosing reasonable sampling directions to fill the multidimensional hypercube cells with as many uniform points as possible. Among them, the Sobol sequence (Bratley and Fox, 2003) has a shorter computational cycle, faster sampling speed, and higher- efficiency in dealing with high-dimensional sequences. Therefore, this paper uses the Sobol sequence to map the initialized populations.

Setting the range of values of the optimal solution to [*x*_*min*_,*x*_*max*_], the Sobol sequence generates the random number *K*_*n*_ ∈ [0,1], then the initial position of the population can be defined as:


(4) $$x_{n}=x_{m\,i\,n}+K_{n}\cdot \left(x_{m\,a\,x}-x_{m\,i\,n}\right)$$

#### Black hole mechanism

The black hole mechanism is taken from the Multiverse Optimization algorithm (MVO). Each candidate solution in the iterative MVO method is a black hole, the ideal universe is a white hole according to the roulette principle, the black hole and the white hole exchange stuff (dimensional replacement), and some of the black holes can travel nearby through wormhole linkages (population optimal vicinity search). It is assumed that wormholes exist between each universe and the optimal universe so that local changes in each universe can increase the universe’s expansion rate through wormholes. The specific mechanism is expressed as follows.

Assume that wormholes exist between each universe and the optimal universe, allowing local variations in each universe to increase the universe’s expansion rate via the wormholes. The mechanism is denoted by Eq. (5)-Eq. (6), where *WEP* is the proportion of wormholes in the universe. *TDR* is the distance between the optimal universe and the object passing through the wormhole transformation.

when *r*_2_*WEP*,


(5) $$x_{i\,j}=\left\{ \begin{array}{c} X_{j}+T\,D\,R\cdot \left(\left(b_{u,j}-b_{l,j}\right)\cdot r_{4}+b_{l,j}\right)\,r_{3}<0.5\\ X_{j}-T\,D\,R\cdot \left(\left(b_{u,j}-b_{l,j}\right)\cdot r_{4}+b_{l,j}\right)\,r_{3}\geq 0.5 \end{array}\right.$$

when *r*_2_≥*WEP*,


(6) $$x_{i\,j}=x_{i\,j}$$

where *X_j_* is the current optimal universe, *b*_*u,j*_ and *b*_*l,j*_ are the boundary values of the *j*th variable, respectively; *r*_2_,*r*_3_,*r*_4_ are random numbers between 0 and 1; The values of *WEP* and *TDR* are shown in Eq. (7)- Eq. (8).


(7) $$W\,E\,P=W\,E\,P_{m\,i\,n}+l\cdot \left(\frac{W\,E\,P_{m\,a\,x}-W\,E\,P_{m\,i\,n}}{L}\right)$$

(8) $$T\,D\,R=1-\frac{l^{1/p}}{L^{1/p}}$$

where *WEP*_*max*_ and *WEP*_*min*_ are the boundary values of *WEP*, *WEP*_*min*_ = 0.2 and *WEP*_*max*_ = 1; *l* is the current iteration number; *L* is the maximum number of iterations; *p* is a constant algorithm with a default value of 6.

#### Proposed SBCS

**FIGURE 2:**
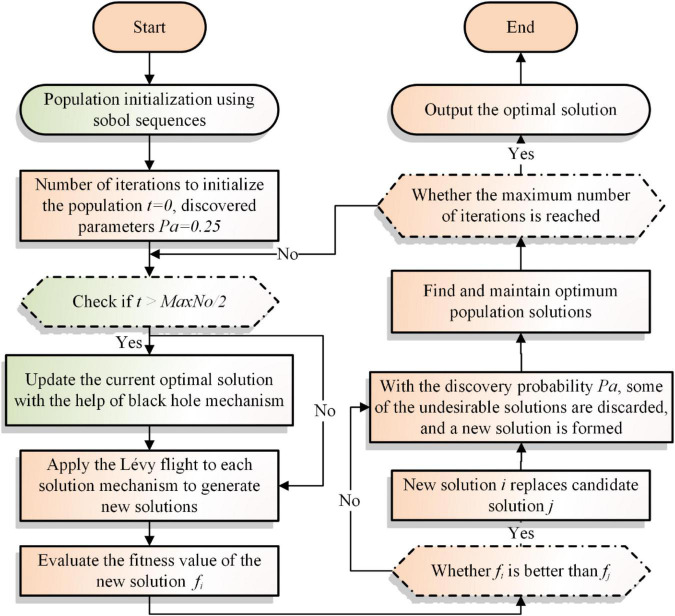
Flowchart of SBCS.

In order to improve the CS algorithm’s early-stage search capacity and late-stage convergence capability, the sobol initialization sequence and the black hole mechanism are added in this section. To begin with, the random starting technique of CS is replaced with the sobol low differentiation sequence in an effort to accelerate the early search phase of the algorithm and identify a high-quality solution more rapidly. Then, the optimal solution is found using the Lévy flight update strategy of the CS algorithm itself. Finally, the black hole mechanism is added at the late iteration stage to work with the CS update method to jointly identify the best solution and improve the algorithm’s convergence ability. SBCS pseudo-code and flowchart are also illustrated in Algorithm 2 and Figure 2.

**Algorithm 2:** Pseudo-code of SBCS.

Population and objective function initialization using sobol sequences *f*(*x*),*x* = (*x*_1_,*x*_2_,*x*_3_,⋯,*x*_*d*_)*T* Initialize the number of iterations*t* = 0, discovered parameters *P*_*a*_ = 0.25 Initialize the solution $x_{i}^{0}$ of the overall *N* solutions, (*i* = 1,2,…,*N*) **While** *t* ≤ *Maximum number of iterations*(*MaxNo*) **If** *t* > *MaxNo*/2 Update the current optimal solution with the help of the black hole mechanism **End If** Using Lévy flight Update all search agents $x_{i}^{t}$ in the population Evaluate the new solution *x^t^*_*new,i*_ for its fitness value *f^t^*_*new,i*_ **If** the new solution has a better fitness value Replace the old solution $x_{j}^{t}$ with the updated solution *x^t^*_*new,i*_ **End If** Discard some poor-quality solutions by probability *P_a_* and replace them with random ones Increase in the number of iterations *t* = *t* + 1 **End While** Return the best solution

The introduced Sobol sequence, the roulette selection mechanism, the rapid sorting algorithm, and the determination of fitness values are the primary components of SBCS’ complexity. First, the Sobol sequence has a computational complexity of *O*(l*n*d + l*n^2^). Then, in two extreme circumstances, the computing complexity of the roulette selection mechanism is *O*(*n*)*andO*(*logn*). The rapid sorting algorithm has a best-case computational complexity of *O*(n*logn) and a worst-case computational complexity of *O*(*n*^2^), respectively. The fitness value calculation has a computational complexity of *O*(n*logn). Finally, the SBCS algorithm’s total complexity is *O*(*SBCS*) = *O*((*d* + *n* + d*logn)*l*n).

### The proposed SBCS-KELM model

#### Discretization

The SBCS algorithm is made to solve continuous optimization issues with real numbers, whereas the feature selection approach is recognized to be a method utilized for binary optimization problems. Therefore, a discrete operation must be performed to transform real values to binary values before the SBCS algorithm can perform the feature selection task. This paper proposes a binary version of SBCS (bSBCS) based on continuous SBCS. First, bSBCS fixes the search range of the solution between [0, 1], i.e., the feature selection problem is regarded as a constrained optimization problem. Then, the searched solution is transformed into a real number of 0 or 1 by the S-type function transformation method, where a solution of 1 means this feature is selected by the model, and a solution of 0 means it is not selected. Eq. (9) displays the solution space’s binary representation.


(9) $$X_{d}\,\left(t+1\right)=\left\{ \begin{array}{cc} 1, & \mathrm{sigmoid}\,\left(X_{d}\,\left(t\right)\right)\geq \mathrm{rand}\\ 0, & \mathrm{otherwise} \end{array}\right.$$

where *X*_*d*_(*t* + 1) is the solution after the t-th binary solution is being updated, and rand is a random integer in the range [0, 1]. The exact Sigmoid formula is shown in the following equation, Eq. (10), where *x* stands for the outcome of the SBCS iteration.


(10) $$s\,i\,g\,m\,o\,i\,d\,\left(x\right)=\frac{1}{1+e^{-2\,x}}$$

#### Kernel extreme learning machine

ELM has an input layer, a hidden layer, and an output layer, three independent layers, and a single implicit feed-forward neural network. For a given training set {(*x*_*i*_,*t*_*i*_)|*x*_*i*_ ∈ *R^n^*,*t*_*i*_ ∈ *R^n^*}, an activation function *f*(*x*), and a number of nodes in the hidden layer *L*, the ELM regression model, can be expressed as:


(11) $$\begin{array}{c} \sum_{t=1}^{L}\beta_{t}\,f\,\left(a_{t}\,x_{1}+b_{t}\right)=t_{1}\\ \sum_{i=1}^{L}\beta_{i}\,f\,\left(a_{t}\,x_{2}+b_{i}\right)=t_{2}\\ \vdots \\ \sum_{i=1}^{L}\beta_{i}\,f\,\left(a_{t}\,x_{k}+b_{t}\right)=t_{k} \end{array}$$

where *a*_*i*_,*i* = 1,…,*L* is the input weight, *b*_*i*_,*i* = 1,…,*L* is the bias, and *k* is the number of samples, the above equation can be rewritten as:


(12) $$T_{k}=H_{k}\,\beta_{k}$$

the neuron matrix *H_k_*, which has the following expression:


(13) $$H_{k}=\left[\begin{array}{ccc} f\,\left(a_{1}\,x_{1}+b_{1}\right) & \cdots & f\,\left(a_{L}\,x_{1}+b_{L}\right)\\ f\,\left(a_{1}\,x_{2}+b_{1}\right) & \cdots & f\,\left(a_{L}\,x_{2}+b_{L}\right)\\ & \vdots & \\ f\,\left(a_{1}\,x_{k}+b_{1}\right) & \cdots & f\,\left(a_{L}\,x_{k}+b_{L}\right) \end{array}\right]$$

The output layer matrix β_*k*_ is represented by the notation:


(14) $$\beta_{k}={\left[\beta_{1},\beta_{2},\cdots ,\beta_{k}\right]}^{\text{T}}$$

The output layer matrix, *T_k_*, may be written as follows:


(15) $$T_{k}={\left[t_{1},t_{2},\ldots ,t_{k}\right]}^{\text{T}}$$

The output weights can be obtained by solving Eq. (16).


(16) $$\beta_{k}=H_{k}^{T}\,{\left(H_{k}\,H_{k}^{T}\right)}^{-1}\,T_{k}$$

The regularized least squares solution of β is obtained, and a regularization factor *C* is added to improve generalization. Its expression is:


(17) $$\beta_{k}=H_{k}^{T}\,{\left(I/C+H_{k}\,H_{k}^{T}\right)}^{-1}\,T_{k}$$

Thus, the extreme learning machine prediction model can be expressed as:


(18) $$t=\sum_{i=1}^{L}\beta_{i}\,f\,\left(a_{i}\,x+b_{i}\right)$$

Kernel functions are used in an ELM algorithm’s implicit layer to replace the feature mapping in a technique known as KELM. The kernel function works by replacing the inner product operation in the new, high-dimensional space with the kernel function operation from the old space when the input training data is mapped into it.

The kernel matrix Ω_ELM_ in the KELM algorithm is as follows.


(19) $$\left\{ \begin{array}{c} \Omega_{\text{ELM}}=H\,H^{T}\\ \Omega_{\text{ELM}}\,\left(i,j\right)=h\,{\left(x_{t}\right)}^{*}\,\,h\,\left(x_{j}\right)=K\,\left(x_{i},x_{j}\right) \end{array}\right.$$

where *x_i_* and *x_j_* are the input vectors of the samples and *K*(*x*_*i*_,*x*_*j*_) is the kernel function, and the radial basis kernel function with strong localization and good generalization is selected in this paper.


(20) $$K\,\left(x_{i},x_{j}\right)=e\,x\,p\,\left(-\frac{{||x_{i}-x_{j}||}^{2}}{\gamma^{2}}\right)$$

where γ is the kernel parameter.

From the above equation, the output function of KELM can be expressed as


$$\hat{y}=h\,\left(x\right)\,\beta =h\,\left(x\right)\,H^{T}\,{\left(H\,H^{T}+\frac{I}{C}\right)}^{-1}$$

$$Y={\left[\begin{array}{c} K\,\left(x,x_{1}\right)\\ \vdots \\ K\,\left(x,x_{N}\right) \end{array}\right]}^{T}\,{\left(\Omega_{E\,L\,M}+\frac{I}{C}\right)}^{-1}\,Y$$

#### Proposed SBCS-KELM

**FIGURE 3:**
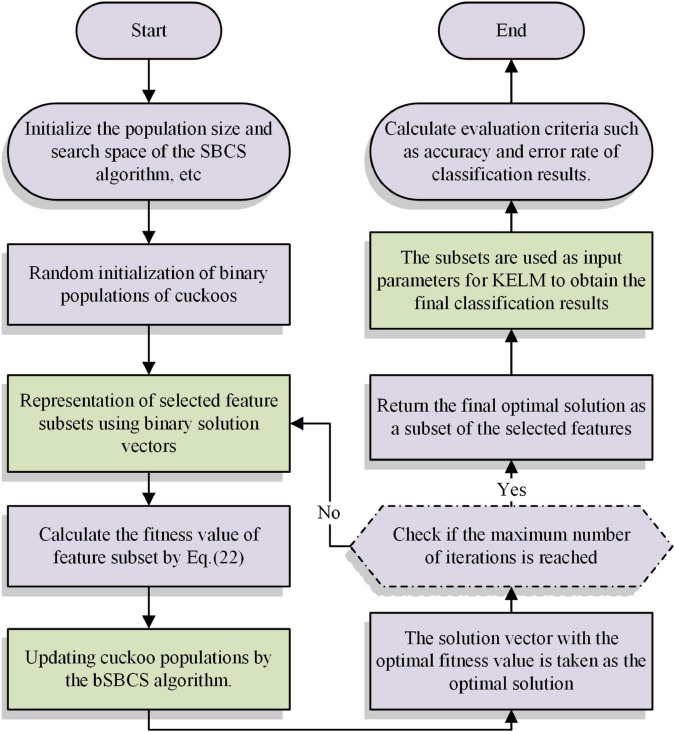
Flow chart of bSBCS-KEML model.

The SBCS-KELM feature selection model, which attempts to filter the important characteristics to aid in medical diagnosis, is built in this part by combining the SBCS algorithm with the KELM. The main strategy for creating models is to use the binary SBCS (bSBCS) algorithm to obtain the best KELM solution. The optimum solutions provided by the bSBCS are then classified using the KELM to improve the model’s classification accuracy and efficiency. The goodness of the solution vector obtained by the bSBCS algorithm needs to be judged by Eq. (22). The bSBCS-KEML method’s main steps are shown in Figure 3.


(21) $$\mathrm{Fitness}=\alpha \cdot \mathrm{error}+\beta \cdot \frac{\left|R\right|}{\left|D\right|}$$

where |*D*| is the number of characteristic characteristics and |*R*| is the number of chosen attributes, and error is the error rate used to measure the classifier’s accuracy. β denotes the length of the chosen characteristics, and α denotes the weight of the mistake; The α = 0.99 and = β 0.01 in this work are the same as in many earlier publications.

## Experimental results and discussion

We undertake benchmark function studies in this part to confirm the overall effectiveness of SBCS. Then, tests based on seven open data sets are carried out to confirm the performance of SBCS on the feature selection issue. This research performs assisted diagnostic studies using PE data gathered from hospitals in order to further certify the efficacy of the SBCS-KELM.

### Benchmark functions comparison experiment

#### Benchmark test experiment setup

As the different algorithms rely on various features and trends, we need to benchmark algorithmic details in computer science to analyze the impact of computational components (Cao Z. et al., 2022; Liu et al., 2022c; Zhang Z. et al., 2022). For the experimental component of the benchmark functions, this work uses 30 benchmark functions from CEC2014 (Liang et al., 2013); Table 4 contains information about benchmark functions F1 through F30.

**TABLE 4:** The 30 benchmark functions in CEC2014.

Class	No.	${\text{F}}_{i}^{*}={\text{F}}_{i}\,\left({\text{x}}^{*}\right)$
Unimodal functions	F1, F2, F3	[100, 300]
Simple multimodal functions	F4, F5, F6, F7, F8, F9, F10, F11, F12, F13, F14	[400, 1600]
Hybrid functions	F17, F18, F19, F20, F21, F22	[1700, 2200]
Composition functions	F23, F24, F25, F26, F27, F28, F29, F30	[2300, 3000]

The benchmark function comparison experiments in this study are being carried out in the same setting and with the same essential parameters, as stated in Table 5, to assure the fairness of the experimental findings. The mean, variance, Wilcoxon signed-rank test, and Freidman test are also employed to examine the final experimental findings in this experiment in order to confirm the validity and reliability of the data. To maintain a standard environment for all of the studies, a Windows Server 2008R2 operating system is used. The device’s main components are a Matlab2017b processor for code execution, an Intel(R) Xeon(R) CPUE5-2660v3 (2.60GHz), and 16 GB of RAM.

**TABLE 5:** Setting the baseline function experiment’s parameters.

Parameter name	Value
Size of the population space	30
Maximum number of evaluations	300,000
Number of tests per algorithm	30

#### Ablation experiments of SBCS

Section “The proposed method” introduces the sobol sequence and the black hole mechanism and both strategies are used to improve the CS algorithm. In this section, ablation experiments are designed to discuss the performance of one strategy alone for improving CS compared to SBCS. Where BCS is the improvement of CS using the black hole mechanism alone, and SCS denotes the improvement of CS using the sobol sequence alone. The algorithms for the comparison experiments include SBCS, BCS, SCS, MVO, and CS.

Table 6 shows the average optimal fitness results of the five compared algorithms and gives the Wilcoxon signed-rank of the compared experiments. The best results are bolded in each column. Where “+/-/ =” indicates that SBCS is better/worse/equal to the other algorithms; “Mean” denotes the average ranking of 30 independent experiments, and “rank” denotes the final ranking. It can be seen that the optimal solution of SBCS is the best among most of the tested functions, followed by BCS with only the black hole mechanism, then SCS with only the sobol sequence, and finally, the two original algorithms MVO and CS. This indicates that either the sobol sequence or the black hole mechanism has an improved effect on CS, and it works best when both are combined. The experiments prove that the improvement of the SBCS algorithm is reasonable and effective.

**TABLE 6:** Average fitness values of ablation experiments and Wilcoxon signed-rank.

Fun	SBCS	BCS	SCS	MVO	CS
F1	**4.4424E+05**	5.2067E+05	9.5602E+05	2.9211E+06	1.0365E+06
F2	1.0000E+10	1.0000E+10	1.0000E+10	**1.7118E+04**	1.0000E+10
F3	3.0000E+02	3.0000E+02	**3.0000E+02**	4.0005E+02	3.0000E+02
F4	**4.0579E+02**	4.1263E+02	4.1002E+02	4.9435E+02	4.1290E+02
F5	5.2006E+02	5.2006E+02	5.2083E+02	**5.2004E+02**	5.2083E+02
F6	6.1465E+02	6.1518E+02	6.2471E+02	**6.0991E+02**	6.2539E+02
F7	7.0000E+02	7.0000E+02	**7.0000E+02**	7.0005E+02	7.0000E+02
F8	**8.2223E+02**	8.2568E+02	8.2723E+02	8.7849E+02	8.3063E+02
F9	1.0148E+03	9.9828E+02	1.0421E+03	**9.9198E+02**	1.0303E+03
F10	1.6721E+03	**1.6585E+03**	2.1439E+03	3.8110E+03	2.1470E+03
F11	3.8569E+03	**3.8534E+03**	4.7021E+03	4.1277E+03	4.5765E+03
F12	**1.2001E+03**	1.2001E+03	1.2007E+03	1.2002E+03	1.2007E+03
F13	**1.3002E+03**	1.3002E+03	1.3003E+03	1.3004E+03	1.3003E+03
F14	1.4002E+03	**1.4002E+03**	1.4003E+03	1.4005E+03	1.4003E+03
F15	1.5078E+03	1.5075E+03	1.5111E+03	**1.5065E+03**	1.5114E+03
F16	1.6114E+03	**1.6113E+03**	1.6123E+03	1.6115E+03	1.6123E+03
F17	3.7964E+03	**3.6920E+03**	3.7147E+03	1.5961E+05	3.8191E+03
F18	1.8841E+03	1.8778E+03	1.8751E+03	9.3280E+03	**1.8707E+03**
F19	**1.9064E+03**	1.9065E+03	1.9083E+03	1.9109E+03	1.9082E+03
F20	2.0467E+03	**2.0464E+03**	2.0610E+03	2.2866E+03	2.0587E+03
F21	3.0855E+03	**3.0254E+03**	3.1611E+03	4.7062E+04	3.1422E+03
F22	2.4359E+03	**2.4270E+03**	2.4365E+03	2.5644E+03	2.4315E+03
F23	**2.5000E+03**	2.6143E+03	2.5000E+03	2.6156E+03	2.6152E+03
F24	**2.6000E+03**	2.6248E+03	2.6000E+03	2.6211E+03	2.6256E+03
F25	**2.7000E+03**	2.7022E+03	2.7000E+03	2.7050E+03	2.7056E+03
F26	2.7003E+03	**2.7002E+03**	2.7003E+03	2.7245E+03	2.7003E+03
F27	**2.9000E+03**	3.1015E+03	2.9000E+03	3.2442E+03	3.1064E+03
F28	**3.0000E+03**	3.6493E+03	3.0000E+03	3.7763E+03	3.7913E+03
F29	**3.1000E+03**	3.9211E+03	3.1000E+03	1.3584E+06	3.8921E+03
F30	**3.2000E+03**	4.2672E+03	3.2000E+03	8.5190E+03	4.8335E+03
+/-/ =	∼	9/1/20	15/1/14	24/5/1	22/2/6
Mean	1.83	2.20	2.80	4.00	3.73
Rank	1	2	3	5	4

#### Comparison of SBCS with well-known peer algorithms

In this section, based on the above-benchmarking functions, we compare SBCS with well-known similar algorithms to demonstrate the superiority of the algorithm performance. Eight algorithms are compared, four of which are the original algorithms SSA, DE, MFO, and WOA, and four of which are the improved algorithms RCACO, ASCA, SMFO, and EWOA. The benchmark experiment findings are shown in Table 7, where AVG and STD stand for the algorithms’ respective mean and variation after 30 separate runs. We can immediately tell by comparing and watching the mean values that SBCS has the least mean value for the majority of the benchmark functions. This demonstrates that when the benchmark functions are optimized using SBCS and related techniques, SBCS delivers considerably higher quality solutions. Additionally, the variation of the ideal solution is lower, demonstrating SBCS’s strong consistency in optimizing the benchmark functions. Additionally, SBCS outperforms hybrid and composition functions, demonstrating the upgraded algorithm’s greater ability to solve challenging situations.

**TABLE 7:** Comparison of SBCS with well-known peer algorithms.

	F1		F2		F3	
	
	AVG	STD	AVG	STD	AVG	STD
SBCS	**3.99175E+05**	**1.27026E+05**	1.00000E+10	**0.00000E+00**	**3.00000E+02**	**1.17819E-06**
SSA	1.61083E+06	6.54909E+05	1.01592E+04	1.00651E+04	1.48124E+03	5.64060E+02
DE	2.14749E+07	5.08759E+06	**5.58050E+02**	6.47059E+02	4.10539E+02	9.81805E+01
MFO	1.10453E+08	8.92773E+07	1.20004E+10	7.15004E+09	9.88925E+04	5.21756E+04
WOA	2.64824E+07	1.31568E+07	4.55291E+06	8.93447E+06	3.81346E+04	2.36943E+04
RCACO	5.73856E+05	4.51449E+05	1.17131E+04	1.21092E+04	3.30835E+03	2.84728E+03
ASCA	2.69513E+08	9.83608E+07	1.91506E+10	3.56913E+09	4.16573E+04	5.79000E+03
SMFO	6.19912E+08	2.75224E+08	4.21752E+10	1.37868E+10	7.79812E+04	6.93897E+03
EWOA	4.53613E+06	3.96256E+06	1.04871E+04	1.02938E+04	5.11429E+03	3.70540E+03

	**F4**		**F5**		**F6**	
	
	**AVG**	**STD**	**AVG**	**STD**	**AVG**	**STD**

SBCS	**4.03441E+02**	**1.24378E+01**	5.20060E+02	**2.17093E-02**	6.14640E+02	2.70545E+00
SSA	4.83651E+02	4.38597E+01	**5.20036E+02**	5.85645E-02	6.19474E+02	3.38094E+00
DE	4.94800E+02	2.95931E+01	5.20567E+02	5.37895E-02	6.19042E+02	**1.67999E+00**
MFO	1.32554E+03	8.74621E+02	5.20337E+02	1.65997E-01	6.24644E+02	3.72619E+00
WOA	5.95082E+02	5.23186E+01	5.20391E+02	1.76874E-01	6.36085E+02	3.24664E+00
RCACO	4.33840E+02	4.46542E+01	5.20669E+02	6.60369E-02	**6.09092E+02**	2.52801E+00
ASCA	1.60225E+03	3.14143E+02	5.20941E+02	5.82806E-02	6.34605E+02	2.48657E+00
SMFO	5.68477E+03	2.00851E+03	5.20963E+02	6.74829E-02	6.37409E+02	2.91633E+00
EWOA	5.23422E+02	4.46295E+01	5.20111E+02	1.14902E-01	6.22330E+02	3.91500E+00

	**F7**		**F8**		**F9**	
	
	**AVG**	**STD**	**AVG**	**STD**	**AVG**	**STD**

SBCS	7.00000E+02	3.47002E-05	8.24417E+02	5.02336E+00	1.01901E+03	2.28138E+01
SSA	7.00012E+02	1.12140E-02	9.01776E+02	2.86079E+01	1.02584E+03	3.80902E+01
DE	**7.00000E+02**	**9.73485E-09**	**8.00825E+02**	**7.33402E-01**	1.01103E+03	**8.32551E+00**
MFO	7.93063E+02	6.99327E+01	9.39879E+02	3.80263E+01	1.11723E+03	5.38819E+01
WOA	7.01016E+02	5.10033E-02	9.93617E+02	3.59734E+01	1.14743E+03	5.47301E+01
RCACO	7.00006E+02	8.96582E-03	8.27930E+02	8.62858E+00	**1.00899E+03**	1.67964E+01
ASCA	8.53007E+02	2.67468E+01	1.04585E+03	2.22212E+01	1.18149E+03	2.16800E+01
SMFO	1.02390E+03	1.24601E+02	1.08273E+03	3.48720E+01	1.20841E+03	2.76460E+01
EWOA	7.00048E+02	5.02529E-02	8.30659E+02	8.86707E+00	1.06871E+03	5.49856E+01

	**F10**		**F11**		**F12**	
	
	**AVG**	**STD**	**AVG**	**STD**	**AVG**	**STD**

SBCS	1.62300E+03	2.14685E+02	**3.89410E+03**	3.95533E+02	**1.20011E+03**	**4.34504E-02**
SSA	4.59087E+03	6.15590E+02	4.72214E+03	8.14252E+02	1.20042E+03	2.40876E-01
DE	**1.02531E+03**	**2.78261E+01**	5.75584E+03	**2.47711E+02**	1.20092E+03	1.47572E-01
MFO	4.35212E+03	9.84691E+02	5.27855E+03	9.16821E+02	1.20042E+03	1.61007E-01
WOA	5.15374E+03	6.63570E+02	6.28644E+03	7.61332E+02	1.20171E+03	5.36213E-01
RCACO	1.94889E+03	3.58625E+02	5.40630E+03	6.88627E+02	1.20117E+03	2.91180E-01
ASCA	6.91363E+03	5.14445E+02	8.32033E+03	2.72372E+02	1.20255E+03	2.66478E-01
SMFO	7.44984E+03	5.49374E+02	8.41713E+03	6.96626E+02	1.20231E+03	5.85183E-01
EWOA	1.73405E+03	3.11883E+02	4.62870E+03	5.32427E+02	1.20036E+03	1.45444E-01

	**F13**		**F14**		**F15**	
	
	**AVG**	**STD**	**AVG**	**STD**	**AVG**	**STD**

SBCS	**1.30024E+03**	**4.80413E-02**	**1.40020E+03**	**2.96041E-02**	**1.50745E+03**	1.87662E+00
SSA	1.30054E+03	1.31869E-01	1.40032E+03	1.40390E-01	1.50900E+03	3.03443E+00
DE	1.30035E+03	4.89097E-02	1.40034E+03	9.00561E-02	1.51190E+03	**8.71991E-01**
MFO	1.30258E+03	1.38356E+00	1.43133E+03	1.71732E+01	9.99364E+04	2.47907E+05
WOA	1.30054E+03	1.44524E-01	1.40026E+03	6.09698E-02	1.57350E+03	2.57744E+01
RCACO	1.30040E+03	7.56017E-02	1.40044E+03	1.99739E-01	1.51229E+03	1.87747E+00
ASCA	1.30314E+03	4.55427E-01	1.44921E+03	7.97005E+00	6.17711E+03	4.19879E+03
SMFO	1.30545E+03	7.72027E-01	1.54165E+03	3.94830E+01	5.06042E+04	5.03831E+04
EWOA	1.30048E+03	9.13298E-02	1.40031E+03	1.29843E-01	1.51910E+03	6.57341E+00

	**F16**		**F17**		**F18**	
	
	**AVG**	**STD**	**AVG**	**STD**	**AVG**	**STD**

SBCS	**1.61118E+03**	4.46582E-01	**3.71383E+03**	**3.07160E+02**	**1.88279E+03**	**1.64880E+01**
SSA	1.61169E+03	6.06837E-01	1.37264E+05	1.00545E+05	7.83598E+03	6.38437E+03
DE	1.61153E+03	2.91654E-01	1.50970E+06	7.18213E+05	8.42411E+03	5.24243E+03
MFO	1.61291E+03	4.90368E-01	3.96263E+06	6.80645E+06	2.18931E+07	8.45145E+07
WOA	1.61261E+03	4.78657E-01	3.43294E+06	2.07061E+06	1.18917E+04	2.95706E+04
RCACO	1.61151E+03	5.78360E-01	1.98567E+05	2.46322E+05	1.55984E+04	5.05022E+04
ASCA	1.61276E+03	**2.03719E-01**	7.04244E+06	4.67239E+06	1.66674E+08	9.56279E+07
SMFO	1.61240E+03	3.31572E-01	4.68655E+07	3.68874E+07	1.24307E+09	1.23023E+09
EWOA	1.61176E+03	5.28520E-01	1.10503E+06	9.96105E+05	7.45030E+03	4.30001E+03

	**F19**		**F20**		**F21**	
	
	**AVG**	**STD**	**AVG**	**STD**	**AVG**	**STD**

SBCS	**1.90627E+03**	7.84254E-01	**2.04573E+03**	**1.50583E+01**	**3.07443E+03**	**2.12093E+02**
SSA	1.91487E+03	2.61475E+00	2.35870E+03	9.50490E+01	5.94502E+04	3.32592E+04
DE	1.90828E+03	**6.59275E-01**	4.79137E+03	1.39744E+03	2.71506E+05	1.76792E+05
MFO	1.95308E+03	5.68659E+01	8.53833E+04	1.37147E+05	5.38854E+05	8.18711E+05
WOA	1.95573E+03	4.02214E+01	2.62512E+04	2.24576E+04	1.50069E+06	1.56997E+06
RCACO	1.90811E+03	1.70117E+00	2.64505E+03	5.11652E+02	1.07463E+05	9.69032E+04
ASCA	1.99938E+03	2.22487E+01	1.65960E+04	8.42374E+03	2.06236E+06	9.49707E+05
SMFO	2.18872E+03	9.12533E+01	1.24262E+05	1.52568E+05	1.45201E+07	1.05100E+07
EWOA	1.92037E+03	2.88502E+01	4.00504E+03	2.80667E+03	5.03243E+05	3.68382E+05

	**F22**		**F23**		**F24**	
	
	**AVG**	**STD**	**AVG**	**STD**	**AVG**	**STD**

SBCS	2.42390E+03	8.46859E+01	**2.50000E+03**	**0.00000E+00**	**2.60000E+03**	**0.00000E+00**
SSA	2.60150E+03	1.46668E+02	2.61528E+03	3.36323E-02	2.63967E+03	8.24149E+00
DE	**2.37501E+03**	**7.32985E+01**	2.61524E+03	1.38756E-12	2.62591E+03	2.75053E+00
MFO	3.06082E+03	2.70122E+02	2.66584E+03	4.79688E+01	2.68586E+03	4.57496E+01
WOA	2.93421E+03	2.60200E+02	2.63271E+03	7.00887E+00	2.60664E+03	4.42408E+00
RCACO	2.47735E+03	1.84292E+02	2.50002E+03	1.54335E-02	2.60008E+03	2.64018E-02
ASCA	3.05520E+03	1.13123E+02	2.50000E+03	1.94335E-02	2.60009E+03	5.36418E-02
SMFO	3.51479E+03	6.39575E+02	2.50000E+03	0.00000E+00	2.60000E+03	1.80988E-05
EWOA	2.74810E+03	1.97950E+02	2.61536E+03	3.80368E-01	2.60399E+03	9.07367E+00

	**F25**		**F26**		**F27**	
	
	**AVG**	**STD**	**AVG**	**STD**	**AVG**	**STD**

SBCS	**2.70000E+03**	**0.00000E+00**	**2.70025E+03**	5.91053E-02	**2.90000E+03**	**0.00000E+00**
SSA	2.71146E+03	3.20957E+00	2.70049E+03	1.11873E-01	3.40401E+03	1.50584E+02
DE	2.70690E+03	9.08146E-01	2.70033E+03	**4.49416E-02**	3.17606E+03	6.46221E+01
MFO	2.71585E+03	8.71755E+00	2.70609E+03	1.93288E+01	3.66723E+03	1.59227E+02
WOA	2.71595E+03	1.61762E+01	2.70044E+03	1.11268E-01	3.81035E+03	3.50844E+02
RCACO	2.70000E+03	2.74808E-04	2.71029E+03	3.04139E+01	2.91042E+03	5.70726E+01
ASCA	2.70000E+03	1.26747E-04	2.70934E+03	2.46480E+01	2.90000E+03	1.98772E-04
SMFO	2.70000E+03	0.00000E+00	2.74357E+03	4.38132E+01	2.90000E+03	0.00000E+00
EWOA	2.71448E+03	6.66091E+00	2.71715E+03	3.77773E+01	3.61099E+03	2.52094E+02

	**F28**		**F29**		**F30**	
	
	**AVG**	**STD**	**AVG**	**STD**	**AVG**	**STD**

SBCS	**3.00000E+03**	**0.00000E+00**	**3.10000E+03**	**0.00000E+00**	**3.20000E+03**	**0.00000E+00**
SSA	3.85152E+03	1.70179E+02	1.88094E+06	4.98070E+06	1.07132E+04	3.30284E+03
DE	3.64251E+03	2.18980E+01	5.87650E+03	4.37159E+03	6.45310E+03	1.19031E+03
MFO	3.93637E+03	2.08812E+02	3.20406E+06	4.06822E+06	5.79458E+04	4.47468E+04
WOA	4.86940E+03	5.85371E+02	7.49485E+06	4.25821E+06	8.63277E+04	6.10417E+04
RCACO	3.02400E+03	1.31439E+02	1.45688E+06	3.30082E+06	7.48349E+03	4.12680E+03
ASCA	3.00000E+03	3.14331E-03	1.82944E+06	6.94308E+06	1.18203E+05	1.50916E+05
SMFO	3.00000E+03	0.00000E+00	3.10179E+03	9.81905E+00	9.03969E+05	7.06750E+05
EWOA	4.29024E+03	2.96948E+02	4.33165E+06	4.40192E+06	9.10073E+03	2.24082E+03

**FIGURE 4:**
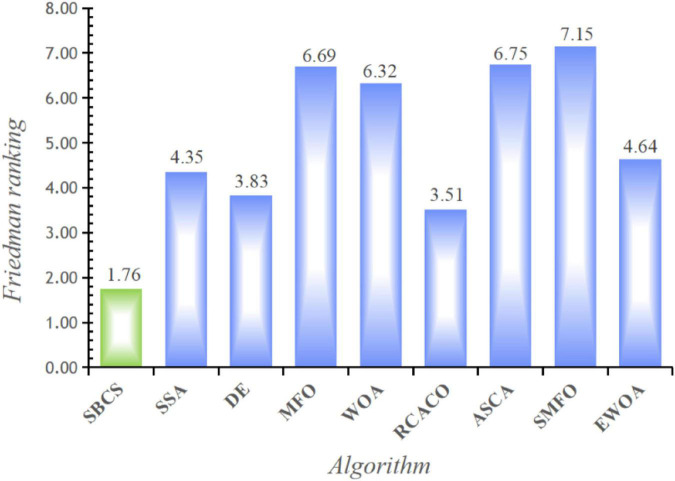
Comparison results of SBCS and other well-known algorithms.

This study examines the experiment’s findings using the Wilcoxon signed-rank test and the Freidman test in order to confirm the statistical significance of the benchmark function experiment and the relative optimization performance of the SBCS algorithm. Based on the Wilcoxon signed-rank test, Table 8 compares the performance of the SBCS with that of other well-known algorithms. A value of “+” indicates that the SBCS outperforms other algorithms, a value of “=” indicates that it performs nearly as well as other algorithms, and a value of “–” indicates that it performs less well than other algorithms. On the 30 benchmark function trials, it is immediately obvious that SBCS is superior to at least 24 other functions and is rated first in this comparison. Figure 4 displays the results of the Freidman test. As can be observed, this technique test still clearly favors the SBCS algorithm. In conclusion, it is possible to infer that SBCS is a great improvement algorithm.

**TABLE 8:** Performance ranking of SBCS with other well-known algorithms.

Algorithm	+/-/ =	Mean-level	Rank
**SBCS**	**∼**	**1.43**	**1**
SSA	26/2/2	4.03	4
DE	24/5/1	3.53	2
MFO	29/0/1	6.80	7
WOA	29/1/0	6.53	6
RCACO	24/2/4	3.67	3
ASCA	30/0/0	6.87	8
SMFO	25/0/5	7.27	9
EWOA	27/1/2	4.73	5

**FIGURE 5:**
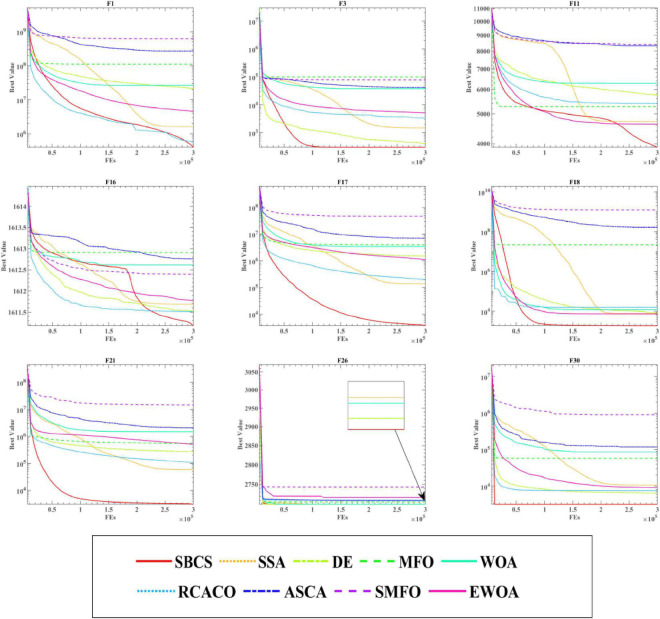
Convergence curves of SBCS and other well-known algorithms.

This experiment shows the solutions produced throughout the iterations of the aforementioned SBCS and the other eight algorithms into curves to further highlight the benefits of SBCS. These four types are addressed in Figure 5 since the 30 benchmark test functions are split into four categories in total: simple multimodal functions, composition functions, hybrid functions, and unimodal functions. Where “FEs” is the quantity of evaluations and “Best Value” denotes the current optimum fitness value. It can be seen that SBCS is a little slower in the beginning of the function curve on functions F1, F3, and F16, but the final convergence is more accurate. What’s more, the convergence images of SBCS have obvious inflection points in the convergence process on functions F11 and F16, which indicates that the algorithm has escaped the trap of local optimum in the stage to achieves higher convergence accuracy. Finally, SBCS has better convergence accuracy than other algorithms for functions F17, F18, F21, F26, and F30, both in the early search phase and in the late convergence. To sum up, it can be concluded that SBCS is an excellent and enhanced algorithm by analyzing the experimental results of SBCS compared with other well-known algorithms.

### Feature selection experiments

The evaluation standards for the feature selection experiments are first described in this section. The effectiveness and generalizability of SBCS to handle feature selection issues are then demonstrated through feature selection experiments on open datasets. Finally, the five key features are chosen using SBCS in a real PE problem.

#### Feature selection experimental setup

We assess the SBCS-capacity KELM’s for classification in the feature selection experiments using the five conventional metrics of sensitivity, classification accuracy (ACC), precision, F-measure, and MCC. The evaluation metrics for classifier experiments are described in detail below.

The performance of binary classification models is typically assessed using 4 main criteria, which include the following:

(1) True Positive (TP): The sample is correctly identified by the classifier, and the sample is regarded as positive.
(2) False Positive (FP): The classifier incorrectly classifies the outcome and believes the sample to be positive when in fact the sample is negative.
(3) False Negative (FN): The classifier incorrectly identifies the outcome and interprets the sample as being negative when it is actually positive.
(4) True Negative (TN): The classifier correctly identified the sample and regarded it as a negative sample.

Accuracy indicates the proportion of the number of correctly classified samples.


(22) $$A\,C\,C=\frac{T\,P+T\,N}{T\,P+T\,N+F\,P+F\,N}$$

The percentage of examples classified as positive cases that are actually positive cases is known as precision.


(23) $$P\,r\,e\,c\,i\,s\,i\,o\,n=\frac{T\,P}{T\,P+F\,P}$$

The effectiveness of the binary classification model in recognizing typical occurrences is evaluated using sensitivity.


(24) $$S\,e\,n\,s\,i\,t\,i\,v\,i\,t\,y=\frac{T\,P}{T\,P+F\,N}$$

A weighted average of Precision (P) and Recall (R) is called F-Measure. Recall is a measure of coverage, which counts the number of positive instances categorized as positive; precision is the percentage of positive examples that are really classified as positive.


(25) $$P=\frac{T\,P}{T\,P+F\,P}$$

(26) $$R=\frac{T\,P}{T\,P+F\,N}$$

(27) $$F=\frac{\left(\alpha^{2}+1\right)\,P^{*}\,R}{\alpha^{2}\,\left(P+R\right)},\alpha =1$$

A more balanced measure for evaluating dichotomies is MCC, another machine learning assessment technique. The measure continues to function effectively even when the difference between the two samples is quite significant since this technique incorporates all four classification situations, i.e., true positive, true negative, false positive, and false negative. A correlation coefficient known as the MCC illustrates the relationship between predicted and observed classifications. It accepts values in the range [–1,1], with 1 being the most accurate topic prediction and 0 indicating no prediction. A score of 1 demonstrates that the predicted and actual classifications agree exactly, a value of 0 shows that the prediction is only as accurate as a random guess, and a value of –1 shows that there is no agreement at all between the two.


(28) $$M\,C\,C=\frac{T\,P\times T\,N-F\,P\times F\,N}{\sqrt{\begin{array}{c} \left(T\,P+F\,P\right)\times \left(T\,P+F\,N\right)\times \left(T\,N+F\,P\right)\\ \times \left(T\,N+F\,N\right) \end{array}}}$$

Immediately following, the nine binary algorithms are used to comparison with the bSBCS in the experiments in this paper including bMFO, bGWO, BGSA, BPSO, bALO, BBA, BSSA, bWOA, and bCS. The parameter values shown in Table 9 for each algorithm are identical to the method’s initial parameter values. The population size of the algorithm is universally set to 20 and the dimension value is always the number of features in the dataset due to the nature of the feature selection approach. Among these 9 algorithms, BBA, BSSA, BWOA, bCS, and BMFO are optimization algorithms based on the original algorithm after discretization. Some other scholars articles mention the algorithms bGWO, BGSA, bALO, and BPSO.

**TABLE 9:** Setting the parameters for the optimization algorithms.

Algorithms	bSBCS	bCS	BBA	bMFO	bWOA
**Values**	*pa = 0.25*	*pa = 0.25*	*a = 0.5; r = 0.5*	*a = 2, b = 1*	*a* = [0,2]
**Algorithms**	BPSO	bGWO	BGSA	bALO	BSSA
**Values**	*Max = 0.9, Min = 0.4*	*a* = [0,2]	*wMax = 20; wmin = 1e-10*	∼	∼

#### Public dataset experiments

Seven open datasets from the UCI Machine Learning Repository are used in this section to assess the overall performance of the bSBCS algorithm on the feature selection problem. These seven open datasets are listed in Table 10 along with additional information about them. These datasets will differ greatly in terms of classification type, number of features, and dataset size in order to simulate the feature selection problem in a wide range of scenarios. Different classification types such as BreastEW for dichotomous classification and segment for multiclassification; different number of features such as heart with 14 and semeion with 266; different dataset sizes such as hepatitis with 155 and CTG3 with 2310. The experimental findings’ mean and standard deviation are also contrasted. In order to further illustrate the experimental findings, each algorithm’s outcomes are statistically rated in this study.

**TABLE 10:** Key messages from the seven public datasets.

Datasets	Samples	Features	Classes
BreastEW	569	31	2
Hepatitis	155	20	2
Segment	2310	20	7
Semeion	1593	266	2
CTG3	2126	22	3
Vehicle	846	19	4
Heart	270	14	2

The classification accuracy of the method suggested in this paper is contrasted with that of other feature selection methods in Table 11. bSBCS is first in the ranking, followed by BGSA, and BCS is last. The classification accuracy of bSBCS on the BreastEW, hepatitis, semeion, and heart datasets is all above 94%, according to specific classification results. Even though its classification accuracy on segment, CTG3, and vehicle did not reach 90%, bSBCS had the most significant classification accuracy of all of the feature selection techniques tested.

**TABLE 11:** Comparison results of SBCS and other peers with regard to accuracy.

Dataset	Metric	bSBCS	bMFO	bGWO	BGSA	BPSO	bALO	BBA	BSSA	bWOA	bCS
BreastEW	Avg	**9.8766E-01**	9.8412E-01	9.8239E-01	9.8587E-01	9.8424E-01	9.5952E-01	9.4733E-01	9.8421E-01	9.8587E-01	9.7547E-01
	Std	1.4554E-02	1.5454E-02	**1.1803E-02**	1.3955E-02	2.2520E-02	2.6662E-02	1.6413E-02	1.3042E-02	1.3955E-02	1.8751E-02
hepatitis	Avg	**9.6120E-01**	9.5500E-01	9.3620E-01	9.4865E-01	9.5453E-01	8.1980E-01	8.5846E-01	9.4870E-01	9.4282E-01	9.0417E-01
	Std	3.3484E-02	3.1106E-02	4.1755E-02	**2.7249E-02**	4.4480E-02	3.6743E-02	7.9550E-02	4.0074E-02	4.6582E-02	5.2924E-02
segment	Avg	**8.8788E-01**	8.8615E-01	8.7706E-01	8.8615E-01	8.8355E-01	7.2900E-01	8.1299E-01	8.8528E-01	8.8312E-01	8.6364E-01
	Std	1.9192E-02	1.8258E-02	1.3720E-02	1.3697E-02	1.6884E-02	4.3155E-02	5.5842E-02	1.0254E-02	**7.0692E-03**	2.1232E-02
semeion	Avg	**1.0000E+00**	**1.0000E+00**	9.9749E-01	**1.0000E+00**	**1.0000E+00**	9.8241E-01	9.7992E-01	9.9938E-01	9.9937E-01	9.9121E-01
	Std	**0.0000E+00**	**0.0000E+00**	6.0733E-03	**0.0000E+00**	**0.0000E+00**	8.2839E-03	1.0975E-02	1.9764E-03	1.9889E-03	1.1909E-02
CTG3	Avg	**8.7866E-01**	8.7862E-01	8.7534E-01	**8.7866E-01**	8.7581E-01	8.2832E-01	8.2596E-01	8.7677E-01	8.7723E-01	8.6408E-01
	Std	1.5050E-02	1.6507E-02	1.5517E-02	1.5790E-02	1.8035E-02	1.5061E-02	2.8295E-02	**9.1786E-03**	1.4202E-02	9.2063E-03
vehicle	Avg	**7.9198E-01**	7.8262E-01	7.8254E-01	7.8480E-01	7.8484E-01	6.3285E-01	6.4500E-01	7.9088E-01	7.8618E-01	7.4120E-01
	Std	**2.5414E-02**	3.4452E-02	3.3220E-02	3.6511E-02	2.7257E-02	7.3121E-02	1.0133E-01	3.1566E-02	4.5623E-02	3.2049E-02
heart	Avg	**9.4444E-01**	9.2963E-01	9.1481E-01	**9.4444E-01**	9.4074E-01	8.4444E-01	8.3333E-01	9.3333E-01	9.2593E-01	8.8889E-01
	Std	5.0148E-02	3.6831E-02	3.5136E-02	3.5994E-02	3.9814E-02	6.7168E-02	9.1141E-02	**3.4035E-02**	3.9040E-02	6.2951E-02
	Rank-Avg	1	3.57	7	2.43	3.71	9.43	9.57	4.14	4.71	8
	Rank	1	3	7	2	4	9	10	5	6	8

Because using only accuracy as an evaluation criterion is insufficient, this study also looked at precision rate. The precision rate of the proposed method SBCS is contrasted with that of other feature selection methods in Table 12. The final two rows of the table display the Wilcoxon signed rank test ranking results; bSBCS is first, followed by BGSA, and lastly bCS. The final precision rate of all feature selection algorithms shows that bSBCS has the greatest accuracy rate, suggesting that its classification findings are the most accurate. SBCS has a lower value as well, as can be seen from Std. SBCS has high robustness, as evidenced by this.

**TABLE 12:** Comparison results of SBCS and other peers with regard to precision rate.

Dataset	Metric	bSBCS	bMFO	bGWO	BGSA	BPSO	bALO	BBA	BSSA	bWOA	bCS
BreastEW	Avg	**9.8115E-01**	9.7574E-01	9.7297E-01	9.7845E-01	9.7858E-01	9.4337E-01	9.3473E-01	9.7582E-01	9.7830E-01	9.7040E-01
	Std	2.2037E-02	2.3481E-02	**1.7789E-02**	2.1105E-02	2.7264E-02	3.9169E-02	2.8441E-02	1.9710E-02	2.1342E-02	2.3330E-02
hepatitis	Avg	**1.0000E+00**	**1.0000E+00**	9.8000E-01	**1.0000E+00**	**1.0000E+00**	5.0000E-01	7.3333E-01	**1.0000E+00**	**1.0000E+00**	9.3333E-01
	Std	**0.0000E+00**	**0.0000E+00**	6.3246E-02	**0.0000E+00**	**0.0000E+00**	4.0825E-01	4.3885E-01	**0.0000E+00**	**0.0000E+00**	1.4055E-01
segment	Avg	**8.8788E-01**	8.8615E-01	8.7706E-01	8.8615E-01	8.8355E-01	7.2900E-01	8.1299E-01	8.8528E-01	8.8312E-01	8.6364E-01
	Std	1.9192E-02	1.8258E-02	1.3720E-02	1.3697E-02	1.6884E-02	4.3155E-02	5.5842E-02	1.0254E-02	**7.0692E-03**	2.1232E-02
semeion	Avg	**1.0000E+00**	**1.0000E+00**	9.9726E-01	**1.0000E+00**	**1.0000E+00**	9.8159E-01	9.8088E-01	9.9931E-01	9.9931E-01	9.9115E-01
	Std	**0.0000E+00**	**0.0000E+00**	6.6203E-03	**0.0000E+00**	**0.0000E+00**	8.9403E-03	9.4059E-03	2.1809E-03	2.1960E-03	1.2367E-02
CTG3	Avg	**8.7866E-01**	8.7862E-01	8.7534E-01	**8.7866E-01**	8.7581E-01	8.2832E-01	8.2596E-01	8.7677E-01	8.7723E-01	8.6408E-01
	Std	1.5050E-02	1.6507E-02	1.5517E-02	1.5790E-02	1.8035E-02	1.5061E-02	2.8295E-02	**9.1786E-03**	1.4202E-02	9.2063E-03
vehicle	Avg	**7.9198E-01**	7.8262E-01	7.8254E-01	7.8480E-01	7.8484E-01	6.3285E-01	6.4500E-01	7.9088E-01	7.8618E-01	7.4120E-01
	Std	**2.5414E-02**	3.4452E-02	3.3220E-02	3.6511E-02	2.7257E-02	7.3121E-02	1.0133E-01	3.1566E-02	4.5623E-02	3.2049E-02
heart	Avg	**9.4007E-01**	9.1818E-01	9.2007E-01	9.0461E-01	9.2006E-01	8.7401E-01	8.3482E-01	9.0958E-01	9.2809E-01	8.9710E-01
	Std	6.8672E-02	5.0378E-02	8.8791E-02	**4.7172E-02**	6.4475E-02	8.3513E-02	9.8461E-02	5.6938E-02	6.4764E-02	7.5451E-02
	Rank-Avg	1	3.57	7	2.43	3.71	9.43	9.57	4.14	4.71	8
	Rank	1	3	7	2	4	9	10	5	6	8

#### Pulmonary embolism dataset experiment

In this part, the obtained PE dataset is utilized for feature selection tests to demonstrate the bSBCS-KELM model’s real predictive performance and efficacy for practical medical assistance diagnosis. Furthermore, to demonstrate the performance of the bSBCS-KELM, four traditional evaluation techniques are utilized to fully analyze the model’s classification ability: classification sensitivity, ACC, F-measure, and MCC, in that order. This section compares bSBCS with other classical machine learning classification algorithms in combination, primarily fuzzy k-nearest neighbor (FKNN), k-nearest neighbor (KNN), multilayer perceptron (MLP), and SVM, to demonstrate that the combination of bSBCS and KELM is excellent. This section also examines the performance differences between the proposed model and conventional classification techniques, such as BP, CART, and others. The codes for these classical classifiers and machine learning algorithms, in particular, are embedded in MATLAB, and the number of neurons for the BP and ELM algorithms is 10 and 20, respectively, with the remainder set to default settings. Furthermore, this part compares bSBCS with well-known algorithms like as bMFO and bGWO to indicate that bSBCS is also suited for KELM among swarm intelligence optimization techniques. To create fair and objective results, the classification performance is analyzed using 10-fold cross-validation (CV) analysis, according to machine learning literature.

**FIGURE 6:**
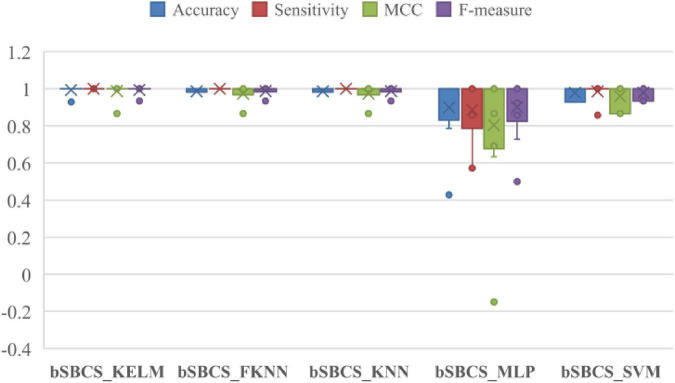
Comparison results of SBCS on five classifiers.

Because various approaches give varied experimental results, a comparative experiment with other classifiers is performed to illustrate the advantages of combining bSBCS with the KELM classifier. Figure 6 depicts the box plots of classifier comparison trials, which reveal that classification performance varies dramatically for various classifiers paired with bSBCS. bSBCS MLP has the lowest accuracy, sensitivity, F-measure, and MCC. bSBCS KNN and bSBCS KELM perform equally in these four categories. A deeper look at the figure represents that bSBCS KELM outperforms all four criteria. As a result, it is possible to speculate that bSBCS KELM is the best classifier among the five.

**FIGURE 7:**
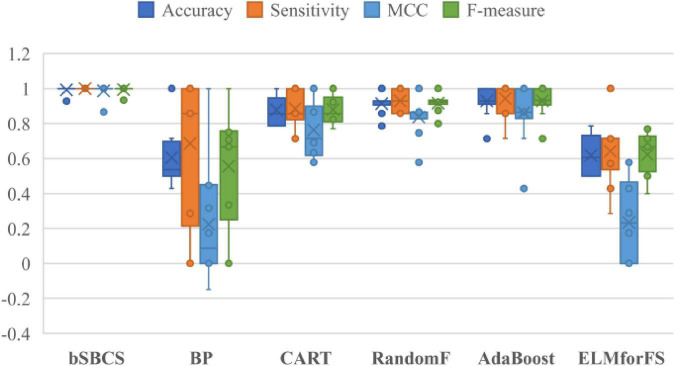
Comparison of bSBCS and other well-known machine learning algorithms.

The influence and effectiveness of feature selection are examined by contrasting the given bSBCS algorithm to those that did not. Figure 7 displays the comparison outcomes of the six classifiers. As shown in the figure, the recommended bSBCS method with feature selection performs better than the original classification strategy without the swarm intelligence algorithm. The bSBCS approach is the best feature selection model for the PE dataset in terms of accuracy, sensitivity, F-measure, and MCC.

We compared the proposed bSBCS method to other generally used feature selection algorithms such as BMFO, BGWO, BGSA, BPSO, BALO, BBA, BSSA, and BWOA to assess its performance on PE datasets. The algorithms’ performance was then evaluated in four areas: classification accuracy, sensitivity, F-measure, and MCC in terms of feature selection.

**FIGURE 8:**
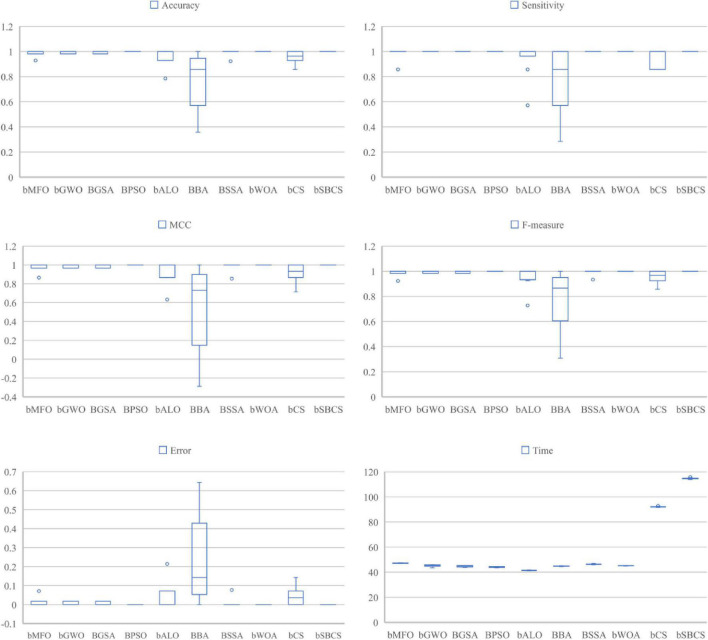
Comparison results of SBCS and other swarm intelligence optimization algorithms.

A box plot of the statistical findings of 10 independent 10-fold cross-validation runs on the dataset is given in Figure 8. SBCS performs well on all six evaluation criteria, according to the results. SBCS has the highest classification accuracy, with sensitivity and F-measure, which represent classification correctness, of 0.98 or higher. Furthermore, the classification accuracy box plots are very concentrated, indicating that the bias of the ten experimental results is small. MCC is also the variable used to indicate the connection between actual and anticipated classes. SBCS’s box plot is the most similar to 1, showing that SBCS can better categorize the PE dataset effectively. Finally, we did the Freidman test on the experimental findings to further demonstrate the outcomes of the comparison between SBCS and other approaches, and the particular ranking results are as follows. In five of the six assessment indicators listed above, bSBCS ranks top, according to the data in Table 13. bSBCS takes longer than other feature selection algorithms since it adds a new mechanism to the original method. bSBCS takes longer than the other algorithms, but it is still within reasonable boundaries. Finally, bSBCS outperforms all other algorithms on the PE dataset.

**TABLE 13:** Freidman test results.

Method		bMFO	bGWO	BGSA	BPSO	bALO	BBA	BSSA	bWOA	bCS	bSBCS
Accuracy	**Avg**	5	4.95	5	4.5	6.8	8.55	4.65	4.5	6.6	**4.45**
	**Rank**	6	5	6	2	9	10	4	2	8	**1**
Error	**Avg**	5	4.95	5	4.5	6.8	8.55	4.65	4.5	6.6	**4.45**
	**Rank**	6	5	6	2	9	10	4	2	8	**1**
Sensitivity	**Avg**	5.3	5.2	4.8	4.8	5.75	7.95	4.8	5.3	6.3	**4.8**
	**Rank**	6	5	1	1	8	10	1	6	9	**1**
MCC	**Avg**	5	4.95	5	4.5	6.8	8.55	4.65	4.5	6.6	**4.45**
	**Rank**	6	5	6	2	9	10	4	2	8	**1**
F-measure	**Avg**	5.05	4.95	4.95	4.5	6.8	8.6	4.45	4.5	6.75	**4.45**
	**Rank**	7	5	5	3	9	10	1	3	8	**1**
Timecost	**Avg**	8	4.7	4.2	2.2	1	3.8	7	5.1	9	**10**
	**Rank**	8	5	4	2	1	3	7	6	9	**10**

Table 14 displays the outcomes of the bSBCS algorithm’s feature selection. The first column displays the number of 10-fold cross-validation folds, while the second displays the number of features that remain after feature selection. Classification accuracy, sensitivity, MCC, and *F*-measure are the final four columns, in that sequence. The chart indicates that the proposed hybrid approach can effectively choose characteristics from a limited collection while retaining good classification accuracy. The results show that the Accuracy value is 99.29%, the Sensitivity value is 98.57%, the MCC value is 0.9866, and the *F*-measure value is 0.9933.

**TABLE 14:** The detailed results obtained by bSBCS.

Fold	Number of features selected	Accuracy	Sensitivity	MCC	F-measure
#1	5	1.0000	1.0000	1.0000	1.0000
#2	9	1.0000	1.0000	1.0000	1.0000
#3	5	1.0000	1.0000	1.0000	1.0000
#4	5	1.0000	1.0000	1.0000	1.0000
#5	6	1.0000	1.0000	1.0000	1.0000
#6	4	1.0000	1.0000	1.0000	1.0000
#7	5	0.9286	0.8571	0.8660	0.9333
#8	7	1.0000	1.0000	1.0000	1.0000
#9	4	1.0000	1.0000	1.0000	1.0000
#10	10	1.0000	1.0000	1.0000	1.0000
Avg.	∼	**0.9929**	**0.9857**	**0.9866**	**0.9933**
Std.	∼	0.0226	0.0452	0.0424	0.0211

**FIGURE 9:**
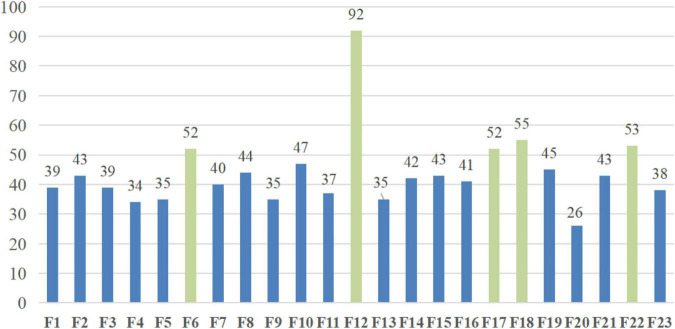
Selected features of the bSBCS method in the 10 times 10-fold CV process.

The precise experimental outcomes of bSBCS on the PE dataset for 10 times 10-fold cross-validation are shown in Figure 9. The PE dataset’s numerous attributes are shown on the figure’s horizontal axis, and the vertical axis shows how often each feature was chosen. As indicated in the diagram, attribute 6, attribute 12, attribute 17, attribute 18, and attribute 22 are mostly selected more than 52 times, while the other attributes are being selected fewer than 47 times. Syncope, SBP, WBC, NEUT%, and SaO2 are the five features. The following section provides a detailed explanation of the experiment findings and how they relate to actual medical applications.

## Discussion

Syncope is linked to a poor prognosis for acute PE, according to earlier research (Goldhaber et al., 1999; Lobo et al., 2006; Lankeit et al., 2013; Keller et al., 2016a; Prandoni et al., 2016; de Winter et al., 2020). According to the ESC, syncope is characterized by transient global cerebral hypoperfusion, which results in transient loss of consciousness (T-LOC). It has a quick onset, a brief course, and a full, natural recovery (Brignole et al., 2018). Uncertainty surrounds the pathogenesis of syncope during PE. According to the available data, a significant pulmonary embolism with pulmonary vascular tree occlusion, right heart dysfunction, decreased cardiac output, hypotension, decreased cerebral blood flow, and ultimately caused syncope is the primary mechanism (Castelli et al., 2003; Theilade et al., 2010; Jenab et al., 2015). A blood clot may also result in arrhythmia if it exits the venous system and remains in the pulmonary circulation. PE syncope may also result from this (Akinboboye et al., 1993; Keller et al., 2015b). The Bezold–Jarisch reflex, as well as a sudden drop in cardiac output, vasodilation, and cardiogenic syncope, may also result from occlusion of the pulmonary artery bed (Mark, 1983; Simpson et al., 1983; Castelli et al., 2003). In general, people who experience cardiac syncope are more likely to pass away from cardiovascular causes (Soteriades et al., 2002). de Winter et al. (2020) discovered through mete analysis that syncope is linked to 4% high short-term mortality and 12% high hemodynamic instability in patients with acute PE. Hemodynamic instability can be used to explain the higher short-term mortality. Additionally, the current risk stratification for pulmonary embolism is based on hemodynamic status (Jaff et al., 2011; Konstantinides et al., 2020). Thus, syncope can be a potent indicator of the severity of a pulmonary embolism because it may reflect hemodynamic instability brought on by right ventricular dysfunction and decreased left ventricular filling.

Systolic blood pressure (SBP) is an important factor in the early risk classification of PE patients, according to current recommendations. While both the ESC recommendations and the AHA statement advocate SBP 90 mmHg as a crucial sign of early mortality from acute PE (Jaff et al., 2011; Konstantinides et al., 2020), the important PE prognosis score, PESI, indicates hypotension with SBP 100 mmHg as an essential predictor of poor prognosis (Wicki et al., 2000; Aujesky et al., 2005). Hemodynamics have a major role in the outcome of an acute PE event, and Keller et al. noted in research with SBP120 mmHg would suggest an elevated risk of mortality in patients with pulmonary embolism (Keller et al., 2015a). Hemodynamics, which may be seen when 30% of the pulmonary artery bed is obstructed by thrombus material, are the major outcome of an acute PE episode. The substantial obstruction of the blood flow to the lung lobes or multiple lungs by PE thrombotic material may result in right heart failure, inadequate blood pressure control (hypotension), and a high risk of short-term death (Grifoni et al., 2000). In conclusion, SBP is a potent predictor of the likelihood of pulmonary embolism.

Acute PE may affect the patient’s heart, lungs, gas exchange, and circulation, which might result in hypoxia. Hypoxia may have physiological effects such tachycardia, dyspnea, dilated blood vessels in the extremities, and increased cardiac output. Additionally, hypoxia-mediated vasoconstriction is one of the causes of acute pulmonary hypertension and a key contributor to acute right heart failure in PE patients (Wang et al., 2019; Labaki and Rosenberg, 2020). Pulse oximetry (SpO2) or arterial blood sample (SaO2) may both be used to assess SO2, which is the ratio of the volume of HbO2 bound to oxygen in the blood to the total volume of bound hemoglobin (Collins et al., 2015). One of the PESI and simplified PESI indicators is SO2. Patients who had an oxygen saturation level of 90% or above did not get a score (Aujesky et al., 2005). In order to guarantee that the real oxygen level stays over 90% for the majority of the time, the British Thoracic Society recommendations advise that the target SO2 for patients with hypoxemia (including PE patients) be greater than 94% (O’Driscoll et al., 2008). The bottom limit of pulse oximetry at sea level, according to Kline et al. (2003), is 94.5%, which may successfully separate PE patients into high-risk and low-risk groups. However, according to Kristen et al., the lowest limit for SO2 should be set at 92.5% (Nordenholz et al., 2011). The investigation by Erol et al. (2018) yielded the best target saturation of 91.5%. So, SO2 has been shown to be a reliable predictor of PE prognosis risk.

White blood cell (WBC) count and neutrophil percentage (NEUT%) are powerful predictors in this model. Several biological explanations exist for the link between increased WBC counts and increased PE mortality. More and more studies have shown that acute PE combined with moderate or severe pulmonary hypertension can lead to the lysis of right ventricular myocytes and the infiltration of inflammatory cells such as neutrophils in humans and rats (Iwadate et al., 2001; Begieneman et al., 2008; Watts et al., 2008). This inflammation can independently magnify the damage (Watts et al., 2010). Therefore, elevated WBC and NEUT% may indicate PE-related right heart dysfunction (Venetz et al., 2013). Some evidence also suggests that WBC counts are correlated with fibrinogen, factor VII, and factor VIII levels (Bovill et al., 1996). Consequently, elevated WBC may be a sign of hypercoagulability, leading to a poor prognosis of PE. The study of Venetz et al. (2013) showed that WBC is an independent risk factor predicting the prognosis of acute PE (Venetz et al., 2013). The study of Jo et al. (2013) also reached a similar conclusion. In addition, the study of Wang et al. (2018) also showed the importance of neutrophils in the prognostic evaluation of PE.

However, this study also has some limitations. First of all, our data set is single-center; we need to conduct a multi-center study to verify this model externally. Secondly, we plan to add more indicators to improve the model’s predictive ability in future research. Finally, we will discuss ways to improve the model performance in many domains, such as information retrieval services (Wu et al., 2020a,2021b), recommender systems (Li et al., 2014, 2017), human activity recognition (Qiu et al., 2022), location-based services (Wu et al., 2020b,2021a), named entity recognition (Yang et al., 2022), clustering of cancer attributed networks (Gao et al., 2021; Wu and Ma, 2022), disease identification and diagnosis (Su et al., 2019; Tian et al., 2020), image denoising (Zhang et al., 2020), tensor completion (Wang W. et al., 2022), structured sparsity optimization (Zhang X. et al., 2022), power flow optimization (Cao X. et al., 2022), colorectal polyp region extraction (Hu K. et al., 2022), and smart contract vulnerability detection (Zhang L. et al., 2022).

## Conclusion and future works

In this paper, we propose a stronger meta-heuristic algorithm SBCS and a KELM model based on SBCS to achieve feature selection for real PE datasets. SBCS is a CS-based algorithm that has been enhanced. The sobol sequence and the black hole mechanism are combined with CS in this work to improve SBCS’s search skills and its capacity to break out of local optimal solutions, enabling SBCS to get higher-quality solutions. First, we do comparative testing on 30 benchmark functions. The findings of the aforementioned compared experiments show that SBCS outperforms CS in terms of search effectiveness and ability to locate high-quality answers. Additionally, a comparison between SBCS and related algorithms shows that SBCS has a stronger overall ability to avoid the local optimum trap and provide better solutions than comparable algorithms. As a result, SBCS is a well-validated and outstanding SIOA. Later, we construct the SBCS-KELM prediction model by discretizing the SBCS algorithm and applying it to the KELM classifier. The model verifies the accuracy and stability of the SBCS-KELM prediction model through the same type classifier experiment, the same type swarm intelligence algorithm experiment, and the same type data set to experiment and taking the sensitivity of classification results, ACC, F-measure, and MCC as experimental indicators. The experimental results show that the five most important characteristics of PE are syncope, SBP, WBC, NEUT%, and SaO_2_. Finally, the detailed discussion of the model shows that the five characteristics are statistically significant, further illustrating its validity and significance for medical diagnosis. The proposed model also has some limitations, as the introduction of sobol sequence and black hole mechanism makes SBCS more complex and time-consuming than the original algorithm. However, this issue will soon be resolved due to the quick development of parallel computing and high-performance computing technology.

SBCS might be used not only for medical diagnosis but also for engineering and artificial neural network optimization in the future. In addition, we will discuss ways to improve SBCS’ performance in many domains.

## Data availability statement

The original contributions presented in this study are included in the article/supplementary material, further inquiries can be directed to the corresponding authors.

## Ethics statement

This study complied with the Helsinki declaration and was approved by the Ethics Committees of The First Affiliated Hospital of Wenzhou Medical University (Wenzhou, China) (Ethical approval code: KY2021-R097).

## Author contributions

HS, ZH, DZ, FY, AH, YF, and YZ: writing—original draft, writing—review and editing, software, visualization, and investigation. YS, PW, HC, and YC: conceptualization, methodology, formal analysis, investigation, writing—review and editing, funding acquisition, and supervision. All authors contributed to the article and approved the submitted version.
